# “Even if I’m undetectable, I just feel like I would die”: a qualitative study to understand the psychological and socioeconomic impacts of the COVID-19 pandemic on women living with HIV (WLWH) in Chicago, IL

**DOI:** 10.1186/s12905-022-01812-z

**Published:** 2022-06-10

**Authors:** Samantha A. Devlin, Amy K. Johnson, Moira C. McNulty, Olivier L. Joseph, André Hall, Jessica P. Ridgway

**Affiliations:** 1 Department of Medicine, University of Chicago, 5837 S. Maryland Avenue, L-038, Chicago, IL 60637 USA; 2 Ann & Robert H. Lurie Children’s Hospital of Chicago, Chicago, IL USA; 3 Feinberg School of Medicine, Northwestern University, Chicago, IL USA

**Keywords:** Women living with HIV, COVID-19, Gender disparities, Unemployment

## Abstract

**Background:**

The COVID-19 pandemic has affected the health and well-being of people worldwide, yet few studies have qualitatively examined its cumulative effects on ciswomen living with HIV (WLWH). We aimed to explore how the pandemic has impacted WLWH, including challenges related to HIV care, employment, finances, and childcare. We also investigated how HIV status and different psychosocial stressors affected their mental health.

**Methods:**

We performed 25 semi-structured qualitative interviews with WLWH regarding the ways in which COVID-19 impacted their social determinants of health and physical well-being during the pandemic. 19 WLWH who received care at the University of Chicago Medicine (UCM) and 6 women who received care at Howard Brown Health, a federally qualified health center (FQHC) in Chicago, were interviewed remotely from June 2020 to April 2021. All interviews were audio recorded and transcribed. Interviews were thematically analyzed for commonalities regarding HIV-specific and general experiences of WLWH during the pandemic.

**Results:**

The majority of participants reported COVID-19 impacted their HIV care, such as appointment cancellations and difficulties adhering to antiretroviral therapy. In addition to HIV care obstacles, almost all participants described perceived heightened vulnerability to or fear of COVID-19. The pandemic also affected the socioeconomic well-being of participants, with reported financial strains and employment disruptions. Some mothers took on additional childcare responsibilities, such as homeschooling. Increased mental health concerns and negative psychological effects from the social isolation associated with the pandemic were also experienced by most participants.

**Conclusions:**

We gained invaluable insight into how WLWH were challenged by and adapted to the COVID-19 pandemic, including its destabilizing effects on their HIV care and mental health. Women described how they undertook additional childcare responsibilities during the pandemic and how their HIV status compounded their concerns (e.g., perceived heightened vulnerability to COVID-19). Strategies to better support WLWH in maintaining their overall health throughout the pandemic include childcare assistance, access to affordable mental health services, support groups, and education from HIV care providers. These findings have significant implications for examining future health crises through the perspective of potential gender inequalities.

## Background

The COVID-19 pandemic has uniquely impacted people living with HIV (PLWH) worldwide [1–8]. PLWH, who are disproportionately impacted by mental health [9–11] and substance use disorders [12, 13], may suffer increased psychosocial burdens as a result of the pandemic. Qualitative studies have demonstrated that PLWH have experienced perceived heightened vulnerability to COVID-19 throughout the pandemic [4, 14–17]. PLWH have also voiced concerns that having COVID-19 may lead to HIV status disclosure [4] and further stigmatization, particularly for Black/African American patients [4, 15, 18].

Additionally, the COVID-19 pandemic has disproportionately impacted the childcare responsibilities and psychological well-being of women. The burden of unpaid care work has increased among women, exacerbating preexisting gender inequalities [19]. Women have assumed almost twice as much of the childcare responsibilities compared to men [20] and have suffered more work disruption than men [21–24]. During the pandemic, women have experienced a higher degree of multifactorial stress when compared to men [25]. Women feel a greater sense of COVID-19 anxiety [26] and may also experience greater physiological and psychological effects from the pandemic [22, 27, 28]. Moreover, a high degree of comorbidity across mood disorders, anxiety disorders, and substance use disorders has been found among women living with HIV (WLWH) specifically [29–31].

Throughout the pandemic, research has focused mostly on gender differences in regard to contracting SARS-CoV-2 and how biological sex may affect COVID-19 outcomes [32–36], with less attention paid to the different social and behavioral consequences emerging between men and women. Several studies have qualitatively examined the effects of the pandemic on peripartum women’s lives, including disruptions to prenatal care, anxiety during pregnancy, and postpartum experiences [37–48]. A few studies have discussed the potential impact of the pandemic on the increased risk of mental illness and domestic violence among WLWH [49, 50] and the challenges associated with taking antiretroviral therapy (ART) during the COVID-19 lockdown among WLWH [51], without conducting an in-depth analysis of the complex intersection of these factors. In this study, we aimed to qualitatively examine how WLWH have been impacted on multiple socioeconomic and health levels throughout the pandemic, including HIV-specific challenges related to retention in HIV care, ART adherence, and perceived COVID-19 vulnerability, as well as general experiences regarding mental health/social isolation, childcare, and financial/employment stability.

## Methods

### Design

We conducted a qualitative study using semi-structured interview guides to facilitate one-on-one interviews with WLWH from June 2020–April 2021. The timeline for data collection allowed for recruitment at two different sites: University of Chicago Medicine (UCM), an academic medical center, and Howard Brown Health (HBH), a federally qualified health center (FQHC) in Chicago. It also allowed for a larger sample size and ensured we reached saturation of themes. Eligible participants included patients who had birth sex and gender identity of female and were living with HIV and received care at either UCM or HBH. Both institutions offer social work, case management, and linkage to behavioral health services and other resources for PLWH. These services were continued or expanded throughout the pandemic for PLWH who needed additional support. Eligibility criteria were confirmed by review of the participant’s electronic medical record (EMR). Research staff called potential participants and informed them of the study. If WLWH were willing to participate, researchers scheduled a time to conduct the interview. Ultimately, twenty-five WLWH consented to participate and were enrolled in the study.

All interviews were conducted via telephone or Zoom by trained research staff in a private and secure location. Participants provided informed consent prior to their participation and verbal permission to audio record their interview. No personally identifiable information (e.g., participant’s name) was collected on the audio recording. WLWH could choose to skip questions or stop their participation at any time, without penalty. Participants received $40 in the form of a Visa gift card, an online code for Amazon, or cash sent through an electronic payment application after completing the interview. Audio recordings were professionally transcribed and uploaded to Dedoose, a qualitative data analysis software, for analysis. This study was approved by the Institutional Review Board at the University of Chicago (approval no. 20-0883) and Howard Brown Health.

### Rigor, validity, and reliability

To establish rigor, we developed our interview questions based on two established conceptual frameworks, minimizing the potential effect of research bias inherent in qualitative methodologies (Table 1). Interview questions were designed to capture information related to psychological and social stressors of the pandemic among WLWH. Questions were developed using the HIV and COVID-19 syndemic model [52] to explore potential social determinants of health among PLWH and the Health Disparity framework [53] to understand factors related to individual, patient-provider, and healthcare factors. Questions assessed the impact of the pandemic on childcare, financial/employment stability, mental health, and retention in HIV care among WLWH.

**Table 1:** Example questions from interview guide

Broad domain of qualitative exploration	Example of exploratory qualitative question	Example of themes emerging from qualitative data	Guiding framework
Retention in care	How has the COVID-19 pandemic impacted your ability to remain in HIV care?	Non-adherence to ART; cancelled or missed HIV care appointments	Health Disparities Framework (Understanding—Patient/individual; Provider; Clinical encounter; Health care system)
Fear of COVID-19	At the beginning of the pandemic, how concerned were you about contracting COVID-19? As the pandemic worsened, did your level of concern change?	Perceived vulnerability to COVID-19 due to HIV status; fear of dying from COVID-19	Syndemic Health Framework (Pandemic-Related Stress)
Mental health effects	How has your mental health been affected?	Increased depression and anxiety; social isolation	Syndemic Health Framework (Loneliness; Social Isolation)
Relationship with provider and healthcare system	How do you think having HIV has impacted your overall experience with the healthcare system during the pandemic?	Stigmatization	Health Disparities Framework (Understanding—Health care system)
Psychosocial stress	Can you tell me how the COVID-19 pandemic has impacted your life in general?	Loss of employment; increased financial and childcare strains	Syndemic Health Framework (Pandemic-Related Stress; Food Insecurity; Poverty; Lack of Social Support)

During data collection and analysis, researchers met routinely to discuss preliminary findings and make iterative improvements to ensure the main research questions were being addressed adequately within the interview responses. Researcher reflexivity was performed during both data collection and analysis, with the goal of highlighting participants’ viewpoints without any interference from our research team’s subjective interpretations or biases.

Transcripts were examined using thematic analysis. A preliminary codebook was developed to organize data into common themes related to experiences and perspectives of WLWH during the pandemic. This codebook underwent several iterative revisions based on emergence of themes during interviews. The finalized codebook was applied by the primary coder to an excerpt of transcripts, and the secondary coders coded a subset of the excerpts selected at random until all coders (N = 3) achieved an interrater reliability of Cohen’s kappa score ≥ 0.80, which indicates substantial agreement between coders on assessing themes [54]. The research team was composed of two Infectious Diseases physicians, a research assistant professor, and three research assistants. Interviews were conducted by a graduate medical student, an undergraduate student, and a clinical research coordinator. The primary coder had extensive experience with conducting qualitative research studies and analyzing qualitative data. All coders who performed data analysis were trained and supported by qualitative research experts. Most divergences occurred due to omission, and upon review and revision by all coders, were quickly rectified to 100% agreement. Codes were then applied to all transcripts and were reviewed by all analysts for consensus.

The research team performed constant comparison by coding each transcript iteratively. We met often to discuss initial codes and to assess trends in coding and potential themes across transcripts. Dedoose was used to identify key themes based on clustering of code application. We ensured that each theme was distinct and had enough supporting data to be considered significant to our findings. Saturation of themes was determined when there was a high prevalence of code clustering and no emergence of new themes. The primary analyst organized the prevalent themes across interviews and presented the findings to the research team for consensus. Relevant quotations were selected to highlight each significant theme.

## Results

Of the total 25 WLWH interviewed, 18 (72.0%) identified as Black/African American; the median age was 39 (range 23–62); and 5 (20.0%) had been diagnosed with COVID-19 prior to their interview (Table 2). Each participant had varying experiences and reactions to the COVID-19 pandemic, but commonalities emerged among WLWH. Four significant themes were elicited and organized into HIV-related experiences during the pandemic (Disruptions to HIV Care; Perceived Heightened Vulnerability to COVID-19) and social/psychological stressors during the pandemic (Childcare and Financial/Employment Strains; Mental Health Concerns). Representative quotations were included to demonstrate the impact of these themes among WLWH.

**Table 2:** Participant demographics (n = 25)

Category	Total (n = 25)
*Age*	
Median	39
Range	23–62
*Race*	
Black/African American	18 (72.0%)
White	3 (12.0%)
Two or more races	1 (4.0%)
Unknown	3 (12.0%)
*COVID-19 diagnosis*	
Yes	5 (20.0%)
No	20 (80.0%)

### HIV-related experiences: disruptions to HIV care; perceived heightened vulnerability to COVID-19

#### Disruptions to HIV care

16 participants reported that the COVID-19 pandemic had disrupted their HIV care (e.g., appointment cancellations, loss of provider, or ART non-adherence). Some participants cited telehealth and prescription delivery as facilitators to retention in care or ART adherence and did not experience any notable changes related to their HIV care. However, many women detailed how the pandemic resulted in HIV care physicians cancelling their appointments and challenges related to ART adherence:

*“I just got denied on my prescription refill, for the first time throughout this whole pandemic. So, I called…to get an appointment, and my doctor has no appointments for the foreseeable future.” (ID 21)*

*“Before, everything [HIV care] was perfect. Everything was fine before. Since the pandemic, I have lost my specialist, so I have to find another one. He’s no longer there. And let’s see, it’s been harder with the visits.” (ID 17)*

*“Not for the better [how the pandemic has impacted retention in HIV care]. Because, like I said, since the corona’s been going on, my numbers [HIV viral load] have gone back up.” (ID 16)*

Participants also mentioned avoiding their in-person HIV care appointments due to fear of getting COVID-19:

*“I’m really afraid, really, to go in and out of hospitals now. So, I cancelled my appointment.” (ID 10)*

Additionally, one woman described how the stress of the pandemic led to inconsistencies in her ART regimen:

*“I did forget once to take my medicine, with everything that going’s on. It’s just been a lot. It’s been a lot for me to process, and a lot to deal with, trying to get into a new routine of things. So, sometimes, I just say, ‘Forget it [taking ART].’” (ID 17)*

Conversely, one woman stated that the pandemic had forced her to prioritize her HIV care. Similarly, another woman credited the COVID-19 pandemic for her decision to seek treatment for her symptoms, which ultimately led to her HIV diagnosis and subsequent linkage to care:

*“It [HIV care] had changed because I’m talking to myself, ‘Don’t you dare miss a dose. Don’t you dare, take it. Go take your medicine. Go eat. Go make sure. You gotta keep your levels up.’ I’m constantly talking to myself.” (ID 11)*

*“Maybe COVID contributed to me finding about this [HIV diagnosis], and not letting this get any further than what it was, and to the point where now I am in - I’m on a treatment program.” (ID 23)*

#### Perceived heightened vulnerability to COVID-19

In addition to HIV care difficulties, 23 participants reported perceived heightened vulnerability to or fear of COVID-19. Seventeen participants directly expressed their concern that living with HIV made them more susceptible to either contracting and/or experiencing worse health outcomes from COVID-19 compared to those not living with HIV:

*“I did have a lot of health scare at the beginning because I thought, because I’m immunocompromised that I would get sick.” (ID 21)*

*“It [HIV] made my fear [of COVID-19] more significant, because my status is not under control.” (ID 13)*

*“I knew for sure that I would get it [COVID-19]. I just knew for sure. I said, ‘I’m gonna get it because I’m fighting this thing [HIV]. It’s [COVID-19] gonna grab me. It’s gonna knock my immune system down.’…I thought for sure, I would be a person that caught it. No doubt, I really thought that.” (ID 11)*

*“I think I have to take more precautions because…I’m already vulnerable because my immune system is not that of an average person. It is already compromised. It’s already fighting against something [HIV]. So, with that being said, I think that makes me more vulnerable [to COVID-19].” (ID 17)*

Although most participants expressed a high level of concern at some point during the pandemic, 5 participants described extreme fear of death and fatalistic thoughts related to acquiring COVID-19:

*“I feel very kind of worried, not kind, very worried about getting it [COVID-19] by touching the wrong thing or you know, just making a mistake. And then, I could probably die. And I feel like I’m more at risk than other people, like even if I’m undetectable, I just feel like I would die.” (ID 18)*

*I’m big scared [of COVID-19], you know? I was just talking about that. I was like, ‘If I get it, I think I’d die.’ Because you know, that affects our immune system, and by me having HIV I’m already fighting that off, you know?” (ID 20)*

*“Those were the thoughts that kept entering my mind that I needed to get that ready [put affairs in order] because I most definitely was on my way out of here [going to die]. Because I’m thinking if I catch it [COVID-19], I’m not gonna live.” (ID 11)*

### Social/psychological stressors—childcare and financial/employment strains; mental health concerns

#### Childcare and financial/employment strains

Of the 18 women who mentioned their children during the interview, 6 assumed additional childcare responsibilities during the pandemic. Some mothers had adult children who no longer lived at home or required care, but many women described how they took on the role of helping their young children navigate the transition from in-person schooling to virtual/remote learning:

*“I’m homeschooling my son because he’s unable to go in. We had the online learning that we were going through, but I have a child with special needs, so he needs more attention than that. So, I’m impacted in that way and I’m not able to go back to work.” (ID 9)*

*“Yeah, I was on [in charge of] childcare. I had to take my son out of daycare and then do home activities with him so that he can keep up when he does go back to school.” (ID 16)*

Of the total 25 women interviewed, 11 reported experiencing increased financial strains as a result of the pandemic. However, most women stated that the pandemic had no impact on their financial stability, with one woman even mentioning that she had recently purchased a home because she had finally achieved sufficient financial security. 13 participants stated that the pandemic impacted their employment situation. Some women reported being able to work from home, which they considered a privilege during the tumultuous economic climate. Other participants reported losing their jobs, being forced to reduce their paid work hours, or increasing their amount of unpaid labor at home, further exacerbating their financial issues:

*“I’m not employed at the time, but as far as financial, yes it [the pandemic] has [impacted finances]. Got behind on my rent and I caught up a little bit, but I’m not quite where I need to be.” (ID 8)*

*“But as far as income, yeah, my income is decreased because I can’t work every day. And so, now I’m, you know, it’s just basically surviving off of unemployment, which sucks.” (ID 24)*

*“Well, for a moment there, I wasn’t working. There was no help for me. Nobody’s said, ‘Well, you can come over here, here’s food over here, here’s somebody to help you with your light and gas bill. Well, here’s somebody to help you with your rent or whatever.’ There was no services…We just sick and we don’t have anything. We just don’t have it. And with a lot of the medications [ART], you have to eat.” (ID 11)*

One woman noted how being unemployed served as a major stressor for her psychosocial health:

*“I lost my job…And it felt like I was going crazy to get – my phone got cut off. You know? So, a lot of little things I’ve been through, to struggle with.” (ID 20)*

#### Mental health concerns

Some women reported that the pandemic either had no effect on their mental health or a positive effect by presenting more time to reflect on and resolve issues. However, 16 participants reported increased anxiety and depression and other forms of psychological distress. Additionally, 17 women reported experiencing negative effects from social isolation at some point during the pandemic.

Women described how their anxiety began to manifest physically, with one stating:

*“I think a couple of days, I was like, ‘I might need some medication for this’…because I think I went really two weeks straight with high anxiety…I even got so bad one day, I thought I couldn’t breathe…I thought I was having trouble breathing. And I had to talk myself off the mountain.” (ID 11)*

A few participants noted how being at home and isolated from others led to increased depressive symptoms. One participant even described how the social isolation resulted in hospitalization:

*“I would say so [mental health has been impacted by the pandemic]. More down than usual. And been at home more. So, it causes my depression to be a little heightened.” (ID 13)*

*“It [the pandemic] takes a toll on your psyche. Um, feeling you know, you get cabin fever or get a little stir-crazy because you feel like the walls are closing in and people who have anxiety being left alone with their thoughts with nothing else to do, mental health is definitely an issue.” (ID 24)*

*“It’s made me a little bit more depressed…Like I said, it [the pandemic] made me go into a depression. I had to go to the psych part of the hospital. Because once I was just in the house just constantly thinking and thinking, I just thought about that, and it just drives me up the wall.” (ID 16)*

The interplay of different factors impacting the mental health of participants—HIV status, perceived heightened vulnerability to COVID-19, and social stressors—was summarized by one woman:

*“It’s my mental health with the anxiety of, I don’t want it [COVID-19], I don’t need it due to a weakened immune system already. I feel like anything and everything can just automatically can attach to me because I already have health issues. I don’t need any more. So, yeah, when I hear more numbers are going up, when I hear more people are dying, when I hear them things, yeah, it affects a little bit of my mental health.” (ID 7)*

## Discussion

Our study is unique in its overarching goal to qualitatively examine the multifactorial impacts of the COVID-19 pandemic on WLWH in the following areas: retention in HIV care, ART adherence, social/psychological stressors, mental health/social isolation, financial/employment stability, and childcare. Prior studies have focused on how the pandemic has affected WLWH’s mental health and safety [49, 50] or their HIV care [51], but we present comprehensive findings on several facets of socioeconomic, mental, and physical wellness that were affected by the pandemic. These data were collected over the course of 10 months from a large academic center and an FQHC in Chicago, demonstrating a lasting understanding of WLWH’s vantage points, including times of both peak COVID-19 incidence and relative stability. As a result, our findings can contribute to the existing literature surrounding the experiences of WLWH during the pandemic.

In our analysis, WLWH reported many commonalities during the COVID-19 pandemic that were related specifically to their HIV status. Many of the women in our study experienced disruptions to their HIV care, leading to fear of attending visits, cancelled appointments, or ART non-adherence, which has been seen in other studies of PLWH during the pandemic [2, 16, 51, 55–59]. WLWH also reported a high degree of perceived increased vulnerability to COVID-19, particularly at the beginning of the pandemic. Women assumed that having HIV would lead to a poor outcome should they get COVID-19, and some participants reported extreme beliefs that they would die from COVID-19. This concern has been voiced by PLWH in other studies as well [4, 15, 60, 61].

In terms of social stressors, a significant number of WLWH in our study reported increased financial/employment strains and childcare responsibilities as a result of the pandemic. Many women in our study were forced to reduce their paid work hours or lost their jobs completely, limiting their ability to pay rent and other bills. We have seen that women not living with HIV have experienced an exacerbation of existing gender disparities throughout the pandemic, including unequal division of childcare work even if men increased their pre-pandemic household contributions, as well as more transitions to unemployment or reduced work hours among women in general [20, 23, 59, 62–68]. Several WLWH undertook additional childcare tasks and reported being responsible for facilitating their children’s remote learning and/or taking care of their kids during the day while childcare centers were closed. These financial impacts, in combination with unanticipated unpaid additional labor at home, were reported to negatively affect the mental health of our participants.

Although PLWH and the general population have experienced psychological distress throughout the pandemic, psychiatric disorders remain prevalent for women and WLWH in particular. Many of our participants reported increased depression or anxiety and negative effects from social isolation due to the pandemic. There has been an excess of suicides among women but not men as a result of the psychological burden of COVID-19 within certain regions of the world [69]. This gender difference in psychological responses to the pandemic is likely due to the heavier toll that COVID-19 has had on women and the lack of sociocultural support rather than gender-based differences in resilience [70].

These comorbidities, in addition to the social challenges imposed by the pandemic, likely complicate clinicians’ ability to provide coordinated HIV care to WLWH. Examining these factors together, the intersectionality of HIV status, gender, finances/employment, and mental health becomes critically important. During the pandemic, women who spent long hours on childcare or housework duties were more likely to report psychological distress; adapting their work patterns further increased this distress [68]. Similarly, WLWH are more likely to experience depression if they are unemployed, have a financial burden, or suffer from a common opportunistic infection [71, 72]. Our qualitative analysis highlights the importance of creating psychosocial interventions that acknowledge how gender-based inequities may contribute to the mental and emotional distress experienced by WLWH. Higher stress, fewer active coping strategies, and less perceived social support have been associated with greater anxiety and depression symptoms among low-income and minority WLWH [73]. This disparity is particularly relevant for our analysis, as a majority of the participants were Black/African American women who lived in low-income and medically underserved areas on the south side of Chicago.

The women in our analysis expressed how the pandemic had impacted their childcare responsibilities, mental health, and perceived vulnerability to COVID-19. Some studies have suggested focusing on intervention strategies that build resilience and alleviate the psychosocial burdens of stigma and depression among WLWH, particularly for those who are marginalized [74]. Based on our findings, we recommend healthcare systems adopt strategies to better support WLWH throughout the pandemic and potential future crises. Efforts should include childcare assistance, access to affordable and quality mental health services, social support groups with other WLWH, and provision of educational materials by trusted HIV care providers to mitigate unfounded concerns of susceptibility to COVID-19.

This study has a few limitations. Participants were recruited from hospital/clinic sites (UCM, HBH), so our results may not transfer to WLWH who are not engaged in care. Nevertheless, we still saw a high degree of negative impacts of the pandemic among our study participants, and it is likely that those who are less engaged in the healthcare system may have similar or even more pronounced socioeconomic or psychological distress compared to the women in our analysis. The vast majority of interviews were conducted before vaccines were widely available and may not be transferable to later stages of the pandemic. However, we saw similarities in responses throughout the entire study period (June 2020–April 2021), indicating that these are enduring struggles and not initial reactions limited to the first few months of the pandemic. Public schools within Chicago remained in remote learning for the entire 2020–2021 school year, so even when the initial lockdown phase of the pandemic was over, the impacts on WLWH with children were pronounced. Additional research should seek to identify long-term effects of the COVID-19 pandemic on WLWH, particularly the potential psychosocial and economic ramifications among minority women. Future studies should also examine healthy coping mechanisms used by WLWH that may build resilience throughout the pandemic and during future crises.

## Conclusions

We gained invaluable insight into how WLWH were challenged by and adapted to the COVID-19 pandemic, including its disruptive effects to their HIV care and mental health. During a period of elevated concern related to COVID-19 and its unique effects on women’s psychosocial and behavioral wellness, those living with HIV have the compounded burden of perceived heightened vulnerability and routine health management. Healthcare systems should utilize strategies and interventions that include childcare assistance, mental health services, support groups, and education from trusted HIV care providers to better support WLWH in social and clinical networks and to mitigate potential gender inequalities that may linger after the pandemic or even reoccur during future health crises.

## Data Availability

There is no contact available to request the data from this study. Data contain protected health information (PHI) and cannot be shared due to confidentiality concerns, as patients may be identifiable.

## Abbreviations

COVID-19: Coronavirus disease 2019
HIV: Human immunodeficiency virus
PLWH: People living with HIV
WLWH: Women living with HIV
SARS-CoV-2: Severe acute respiratory syndrome coronavirus 2
ART: Antiretroviral therapy
UCM: University of Chicago Medicine
HBH: Howard Brown Health
